# Identification of transcription factor genes involved in anthocyanin biosynthesis in carrot (*Daucus carota L*.) using RNA-Seq

**DOI:** 10.1186/s12864-018-5135-6

**Published:** 2018-11-08

**Authors:** Miyako Kodama, Henrik Brinch-Pedersen, Shrikant Sharma, Inger Bæksted Holme, Bjarne Joernsgaard, Tsaneta Dzhanfezova, Daniel Buchvaldt Amby, Filipe Garrett Vieira, Shanlin Liu, M Thomas P Gilbert

**Affiliations:** 1 Natural History Museum of Denmark, University of Copenhagen, Copenhagen, Denmark; 2 Research Centre Flakkebjerg, Department of Molecular Biology and Genetics, Aarhus University, Slagelse, Denmark; 3 Chr. Hansen Natural Colors A/S, Taastrup, Denmark; 4 Department of Plant Protection Biology, Swedish University of Agricultural Sciences, Alnarp, Sweden; 5 BGI-Shenzhen, Shenzhen, 518083 China; 6 NTNU University Museum, Erling Skakkes gate 47A, 7012 Trondheim, Norway; 7 Genome Research and Molecular Biomedicine, Department of Biology, University of Copenhagen, Copenhagen, Denmark

**Keywords:** *Daucus carota L.*, Anthocyanin, RNA-Seq, Differential expression analyses, Transcription factors

## Abstract

**Background:**

Anthocyanins are water-soluble colored flavonoids present in multiple organs of various plant species including flowers, fruits, leaves, stems and roots. DNA-binding *R2R3-MYB* transcription factors, *basic helix–loop–helix* (*bHLH*) transcription factors, and *WD40* repeat proteins are known to form MYB-bHLH-WD repeat (MBW) complexes, which activates the transcription of structural genes in the anthocyanin pathway. Although black cultivars of carrots (*Daucus carota L.)* can accumulate large quantities of anthocyanin in their storage roots, the regulatory genes responsible for their biosynthesis are not well characterized. The current study aimed to analyze global transcription profiles based on RNA sequencing (RNA-Seq), and mine *MYB*, *bHLH* and *WD40* genes that may function as positive or negative regulators in the carrot anthocyanin biosynthesis pathways.

**Results:**

RNA was isolated from differently colored calli, as well as tissue samples from taproots of various black carrot cultivars across the course of development, and gene expression levels of colored and non-colored tissue and callus samples were compared. The expression of 32 *MYB*, *bHLH* and *WD40* genes were significantly correlated with anthocyanin content in black carrot taproot. Of those, 11 genes were consistently up- or downregulated in a purple color-specific manner across various calli and cultivar comparisons. The expression of 10 out of these 11 genes was validated using real-time quantitative reverse transcriptase polymerase chain reaction (qRT-PCR).

**Conclusions:**

The results of this study provide insights into regulatory genes that may be responsible for carrot anthocyanin biosynthesis, and suggest that future focus on them may help improve our overall understanding of the anthocyanin synthesis pathway.

**Electronic supplementary material:**

The online version of this article (10.1186/s12864-018-5135-6) contains supplementary material, which is available to authorized users.

## Background

Anthocyanins are water-soluble, colored flavonoids present in multiple organs of various plant species including flowers, fruits, leaves, stems and roots [1], and are responsible for the red, purple and blue colors [2]. They have many biological roles, including attracting pollinators to flowers and seed dispersers to fruits, as well as conferring defense against plant pathogens, and protection against UV radiation, drought and cold [1, 3–7]. Anthocyanins have also been used as natural replacement of synthetic food colorants [8], and in recent years they have attracted significant attention due to its low toxicity [9] and health-promoting effects, such as protection against cancer, strokes and other chronic human disorders [2, 9, 10].

The biosynthesis of anthocyanins is one of the most extensively studied biosynthetic pathways of secondary metabolites in plants [11, 12]. The pathway is highly conserved across species, involving at least two classes of genes: the structural genes encoding the enzymes that directly participate in the formation of anthocyanins, and the regulatory genes that control the transcription of structural genes [2, 13]. Many of the structural and regulatory genes involved in anthocyanin biosynthesis have been identified, especially in flowers, fruit and leaves [14]. In particular, regulatory genes likely play an important role in determining anthocyanin production, and studies suggest that the pathway is regulated by the interaction of three protein families: DNA-binding *R2R3-MYB* transcription factors, basic helix–loop–helix (*bHLH*) transcription factors, and *WD40* repeat proteins [2, 15, 16]. These regulatory proteins form a ternary *MYB*-*bHLH*-*WD40* (MBW) transcriptional complex, that binds to the promotor of target genes, activating the transcription of structural genes in the anthocyanin pathway [2, 16, 17]. Transcription levels of *MYB* and *bHLH* differ among cell types and in response to environmental conditions [18], while the *WD40* genes are likely to be transcribed constitutively [19]. *MYB* transcription factors often play the key role in regulating anthocyanin production in various plant species, although a few studies have also found some important *bHLH* proteins regulating the pathway [20]. Most of the *MYB*s involved in anthocyanin biosynthesis are positive regulators that enhance the expression of structural genes involved in the pathway. However, negative regulators have also been characterized, such as *VvMYB4* and *VvMYBC2* in grapes [21] and *FaMYB1* in strawberry [22]. Negative regulators interact with *bHLH* protein, thereby competing with the *R2R3-MYB* activators. It is known that different *R2R3-MYB* transcription factors control various flavonoid pathway branches leading to the biosynthesis of anthocyanins, flavonols, and proanthocyanins [23]. *R2R3-MYB* homologs that are involved in anthocyanin biosynthesis have been isolated in various species, including apple (*MdMYB10*) [24] and pear (*PcMYB10*) [25], as well as many other members of the rosaceous family and other species [26].

Carrot (*Daucus carota L.)* is one of the plant species that can accumulate large quantities of anthocyanin in its storage roots [14, 27]. Furthermore, black carrot anthocyanins are known to have higher color stability across a wider range of pH and temperature levels than those from other plant species [28]. This, in combination with high antioxidant activity [29] and levels of nutraceutical components [30], has lead black carrots to be increasingly recognized as an attractive source of anthocyanin. Although Xu et al. [17] have recently discovered that a gene encoding an *R2R3-MYB* protein, *DcMYB6* (designated as *MYB113* in [31]), is involved in regulating anthocyanin biosynthesis in black carrot taproots, the overall regulatory genes responsible for carrot anthocyanin biosynthesis are not well characterized, and key transcription factors such as *bHLH* and *WD40* have not yet been identified. It has been shown that combined induction of *MYB* and *bHLH* proteins leads to high levels of anthocyanins in other plant species [24, 32, 33]; thus, if the aim is to increase anthocyanin biosynthesis in black carrots, it is of particular importance to identify the appropriate *bHLH* partner that forms the right functional MBW complex with *DcMYB6*.

Unlike species in the rosaceous family, previous studies on carrots have attributed differences in anthocyanin accumulation to a handful of structural and regulatory genes that were already isolated and characterized [17, 27]. The current study aimed to analyze global transcription levels, and to mine *MYB*, *bHLH* and *WD40* genes that may function as positive or negative regulators in the carrot anthocyanin biosynthetic pathway. RNA was isolated from differently colored calli, as well as tissue samples from taproots of various cultivars across the course of development. RNA-Seq data were obtained, aligned to the recently published carrot genome [31], and gene expression levels of colored and non-colored tissue and callus samples were compared. Overall we aimed to identify *MYB*, *bHLH* and *WD40* genes that are consistently down- or upregulated in a purple color-specific manner within the various cultivars and different time points sampled. Our results add to the understanding of color variations in black carrot taproots, and allow identification of important regulatory genes that may be involved in anthocyanin biosynthesis.

## Methods

### Experimental design

The analyses relied on three different data sets: 1) calli isolated from taproots of two different carrot cultivars, 2) taproots of a black carrot cultivar, CH5544, sampled at three different time points over the course of development, and 3) taproots of two black carrot cultivars, Nightbird and Superblack, sampled at one time point. RNA was isolated from these samples, and RNA-Seq data were obtained, aligned to the carrot genome [31], and differential expression analyses were performed using colored and non-colored tissue or callus samples. In addition, total anthocyanin content was measured from a subset of calli and taproot samples; the correlation between the anthocyanin content and the level of expression for differentially expressed genes was tested. Finally, genes correlated with anthocyanin content were validated with quantitative reverse transcription polymerase chain reaction (qRT-PCR).

### Plant material and sample information

Four different types of cultivars were used in this study: Danvers, Nightbird, Superblack and CH5544. The seeds of the Danvers cultivar were purchased from Berlin Seeds LLC (item number: 251811505049). The seeds of the Nightbird cultivar were purchased from Plant World Seeds (catalog number: 4750). The CH5544 and Superblack cultivars are breeding lines originating from Chr. Hansen.

**Fig. 1:**
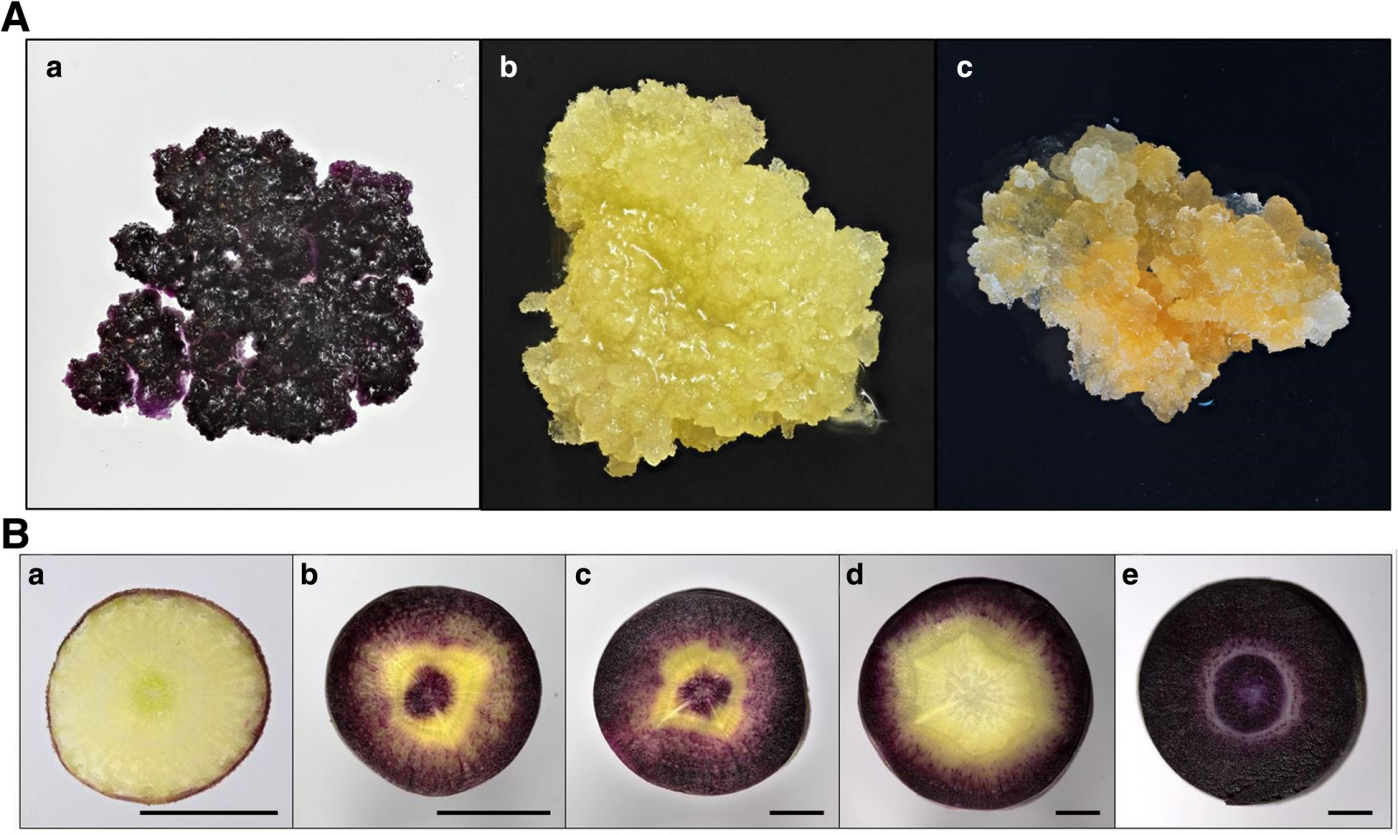
**A** Calli induced on a) CH5544 explants (purple), b) CH5544 explants (white), c) Danvers. **B** Cross section of carrot taproot for a) CH5544 (6 weeks after sowing), b) CH5544 (10 weeks after sowing), c) CH5544 (12 weeks after sowing), d) Nightbird, and e) Superblack. The black bar indicates 1 cm

Calli from the black carrot CH5544 (*Daucus carota ssp. sativus* var. *atrorubens* Alef.) and the orange carrot (*D. carota* var. *sativus*) cultivar “Danvers” were induced on hypocotyls cut into 1 cm explants. The explants were grown on solid B5-medium [34] supplemented with 1 mg/l 2,4-D and 30 g/l sucrose and subcultured every four weeks. For Danvers, only yellow to orange calli were induced (Fig. 1A), whereas the calli induced on CH5544 explants showed a mix of white to purple colors. After two subcultures, the white and the dark purple calli formed on the CH5544 explants (Fig. 1A) were selected and cultured separately for twelve months after culture initiation. Two colonies of purple, white and yellow/orange calli were selected for RNA extraction (Table 1).

**Table 1:** Sample type (callus or tissue), and the age and number of biological samples used for RNA-Seq. Text in parentheses indicates the color of the callus or tissue

Cultivar	Callus	Tissue			Age	N
		outer (epidermis + cortex)	middle (pericycle + endodermis)	inner (xylem and phloem)		
CH5544	× (Purple)	NA	NA	NA	NA	2
CH5544	× (White)	NA	NA	NA	NA	2
Danvers	× (Yellow to orange)	NA	NA	NA	NA	2
CH5544	NA	× (Slightly purple)	× (White)	× (Slightly green)	6 weeks	2
CH5544	NA	× (Purple)	× (White)	× (Purple)	10 weeks	4
CH5544	NA	× (Purple)	× (White)	× (Purple)	12 weeks	2
NightBird	NA	× (Purple)	× (White)	× (White)	12 weeks	2
Superblack	NA	× (Purple)	-	× (Purple)	12 weeks	4

Taproots were obtained from the cultivars, Nightbird, Superblack and CH5544 (*Daucus carota ssp. sativus* var. *atrorubens* Alef.; 2n = 2× = 18; Chr. Hansen A/S, Denmark); seeds of these cultivars were sown in peat (10–12 per 5 L pot) and grown in a greenhouse under 16 h:8 h light:dark photoperiod with ~ 120 μE m^− 2^ s^− 1^ (6480 lx) of light intensity and day/night temperature regimes on ~ 25/20 °C.

The type of taproot tissue and number of individuals sampled from each cultivar are summarized in Table 1. Specifically, taproots were collected from eight CH5544 individuals at three different time points (6, 10 and 12 weeks after sowing) in order to capture genes that are expressed in a purple tissue-specific manner during the emergence to the full development of the purple color. Furthermore, taproots were collected from two Nightbird and 4 Superblack individuals at 12 weeks after sowing.

Each taproot was sliced into a 0.2 cm disks along the horizontal axis, approximately 0.5 cm from the taproot’s top. The taproots of three cultivars exhibited differential pigmentation (Fig. 1B). For the CH5544 cultivar, the epidermis of all carrot discs was darkly pigmented (purple/violet), whereas the cortex was pigmented only in 10 and 12 weeks old roots. The pericycle and endodermis of 6-week-old carrots appeared slightly green, as compared to white/colorless in 10 and 12 weeks old carrots. The vascular taproot tissue was slightly green at 6 weeks and purple in 10 and 12 weeks old taproots. In Nightbird, the epidermis and cortex were darkly pigmented (purple/violet), while the rest of the taproot appeared white. For Superblack, the entire taproot appeared as dark purple, except for endodermis and pericycle.

For CH5544 and Nightbird, the carrot discs were dissected into outer (epidermis + cortex), middle (pericycle + endodermis) and inner (xylem and phloem) tissue samples, whereas for Superblack, the carrot discs were dissected into only outer (epidermis + cortex) and inner (xylem and phloem) tissue samples (Additional file 1). All samples were immediately frozen in liquid nitrogen until homogenization.

### Anthocyanin profiling

A subset of calli and taproot samples was collected for the measurement of anthocyanin content. Approximately 40 g of each sample was coarsely grounded and homogenized in a Waring® two-speed commercial blender (VWR - Bie & Berntsen, Herlev, Denmark) in a 3% sulfuric acid solution (1/1, *w*/w). The homogenate was subsequently mixed with 70% ethanol (1/2, w/w), vortexed and incubated for 1 h at room temperature. The supernatant was separated by centrifuging for 20 min at 4500 rpm and utilized for further analysis using high performance liquid chromatography-diode array detection (HPLC-DAD) and liquid chromatography coupled to (quadrupole) time-of-flight mass spectrometry (LC-MS/Q-TOF) as described in [35].

### RNA extraction and RNA sequencing

Total RNA was extracted from the Nightbird and Superblack samples using 500 mg of frozen tissue per sample, using the Spectrum™ Plant Total RNA Kit (Sigma-Aldrich, USA) supplemented with 0.01 g/mL PVPP (Sigma-Aldrich, USA). Tissues were lysed with the Qiagen TissueLyser (Qiagen, USA) using 3 cycles of 30 Hz for one minute. The RNA extraction was hereafter performed following the manufacturer’s instructions. The total RNA recovered was further purified using the RNeasy Plant Mini Kit (Qiagen, USA) following the manufacturer’s instructions. DNAase treatment was performed to remove remaining DNA using the Ambion DNA-free DNA removal kit (Thermo Fischer Science, USA).

For calli and taproot tissue samples of CH5544, total RNA was extracted using a modified protocol of Direct-Zol™ RNA MiniPrep kit (Zymo Research, USA). Approximately 500 mg of frozen tissue sample was homogenized in a sterilized (RNaseZAP, Sigma-Aldrich, USA) pestle and mortar with 6 mL of TRI Reagent (saturated with Gluta-thiocyanate, Sigma-Aldrich, USA). Total nucleic acid content was extracted by standard PCI (Phenol-Chloroform-Isomyl, Sigma-Aldrich, USA) extraction and precipitated with 96% EtOH (Sigma-Aldrich, USA). The RNA samples were purified with column purification, then subjected to DNAse treatment using the standard kit protocol. The samples were further cleaned and concentrated using the RNA Clean & Concentrator™-5 kit (Zymo Research, USA) following the manufacturer’s instructions, and finally eluted in 100 μl of DEPC (diethyl pyrocarbonate; Sigma-Aldrich, USA) water.

For all samples, the RNA Integrity was verified by electrophoresis in 0.1% DEPC. The RNA concentration was determined using NanoDrop™ 2000 spectrophotometer (Thermo Fisher Scientific, USA). The cDNA libraries were subsequently prepared and sequenced using Novogene’s commercial service (Hong Kong, China) for calli and the CH5544 taproot samples, and BGI (Shenzhen, China) for the Nightbird and Superblack taproot samples. Paired-end 150 bp libraries with an insert size of 200–300 bp were sequenced on an Illumina HiSeq4000 instrument.

### Data filtering and quality control

Sequence quality of raw RNA-Seq data was assessed using FastQC v0.11.3 [36]. Quality trimming was performed using PRINSEQ v0.20.4 [37] to remove base pairs with a Phred score < 20 and trimming of poly-A tails > 8 bp. Sequences shorter than 55 bp, and all unpaired reads were excluded from subsequent analyses. The quality of trimmed sequences was checked again using FastQC v0.11.3.

### Differential gene expression analysis

In order to identify differentially expressed purple color-specific genes, differential gene expression analyses were performed. First, trimmed reads from all samples were aligned to the reference genome published by Iorizzo et al. [31] using STAR aligner [38] with default parameters. Aligned reads were used to generate a gene-specific count matrix across samples using featureCounts [39]. Differential gene expression analyses were performed using several Bioconductor packages: namely, DESeq2 [40], EdgeR using glmLRT and glmQL models [41, 42] and Limma [43]. Genes with an adjusted *P*-value or False Discovery Rate (FDR) < 0.05 found by these packages were considered as differentially expressed. *MYB*-, *bHLH*- and *WD40*-transcription factors are known to play essential roles in the transcriptional regulation of structural genes in anthocyanin biosynthesis. To mine candidate genes for these transcription factors, various color-specific and tissue-specific comparisons were performed to find genes that are differentially expressed in the purple tissue of taproot or callus. In particular, differential expression analyses were performed among the following comparisons: 1) purple, white and orange calli (Table 1), 2) outer purple and middle white tissue of 6, 10 and 12 weeks old taproot from the CH5544 cultivar (Table 1), 3) outer purple, middle white, and slightly green or purple inner tissue of 6, 10 and 12 weeks old taproot from the CH5544 cultivar (Table 1), 4) outer purple, middle white and inner white tissue of the 12 weeks old Nightbird taproot (Table 1), and 5) outer and inner purple tissue of the 12 weeks old Superblack taproot (Table 1).

### Statistical analysis of anthocyanin measurements and candidate genes

The degree of correlation between the total anthocyanin content and transcriptome abundance was examined for the *MYB*-, *bHLH*- and *WD40*-transcription factor genes detected by differential expression analyses based on multiple color- and tissue-specific comparisons. Specifically, Trimmed Mean of M-values (TMM) normalization [44] was performed on gene counts obtained from the 13 samples with measured anthocyanin content. Appropriate Box-Cox power transformation lambda values respectively to each gene were identified using the “boxcox” function implemented in the MASS package [45]. Linear regression was performed for each gene using the TMM normalized gene count and Box-Cox transformed anthocyanin content, with tissue type (callus or taproot) as a covariate to test for an association between the transcriptome abundance and anthocyanin content.

### Quantitative PCR validation

#### cDNA preparation

The cDNA was prepared from the total RNA of all samples except for those from Nightbird and Superblack using SuperScript™ II Reverse Transcriptase (Thermo Fisher Scientific, USA). The standard procedure involved addition of 2 μl of Oligo(dT) primer (500 μg/mL) to 1 μg of eluted total RNA and heating at 72 °C for 5 min. The RNA mix was cooled to 25 °C and 48.5 μl of RT Master mix (according to the manufacturer’s instructions) was added, followed by heating to 42 °C for 45 min and 48 °C for 10 min in a thermocycler (Bio-Rad, USA).

#### Reference gene and primer pair selection

Primers for the 11 consistently up- or downregulated candidate genes were designed based on known carrot sequences (Kodama M, et al.: Genome-wide association analyses reveal candidate genes underlying anthocyanin biosynthesis in carrot (*Daucus carota L.*), In preparation) and sequence stretches present in NCBI Genbank, using Premier primer 5, (PREMIER Biosoft, USA) with amplicon length set between 75 and 153 bp (Additional file 2.1). Glyceraldehylde 3-phosphate dehydrogenase (G3PDH) was selected as reference gene using the primer set derived from [46]. The primer pairs were subsequently analyzed for amplification specificity, efficiency and annealing temperature using endpoint PCR on synthesized cDNA.

#### Quantitative reverse transcription polymerase chain reaction (qRT-PCR) setup

The qRT-PCR experiments were performed on a ViiA 7 Real-Time PCR System (Applied Biosystems, USA) using Power SYBR Green PCR Master Mix (Applied Biosystems, USA). A total reaction volume of 12 μl, containing 1 μl of previously diluted cDNA (1:10), 2.4 μl of gene specific primers (1.5 μM each) and 6 μl of SYBR Green PCR Master Mix was added to MicroAmp Optical 384-well reaction plate (Applied Biosystems, USA) and sealed with MicroAmp Optical Adhesive film (Applied Biosystems, USA). All samples were run in three technical replicates, and no-template controls were included in all plates. The qRT-PCR program was run for 40 cycles, each consisting 15 s at 95 °C and 1 min at 60 °C. The dissociation curve profile was analyzed by including an additional step of 15 s at 95 °C, 1 min at 60 °C and a constantly increasing temperature from 60 to 95 °C. The presence of a single peak from a melting curve from the last amplification cycle and single band in electrophoresis confirmed one single PCR product amplification for each primer pair.

Standard curves for each primer pair were calculated across a 4-fold dilution series (1:1 to 1:64) of pooled diluted cDNA (mix of cDNA from all samples) amplified in triplicate. The PCR efficiency was calculated by the eq. E(%) = (10^−(1/slope)^ − 1) × 100, with the slope of linear regression model fitted over log-transformed data of the input cDNA concentrations versus cycle threshold (Ct) values. G3PDH (AY491512) was found to be most appropriate, with E = 99.90% (Additional file 2.1 and 2.2), thus it was selected as a reference gene. The expression levels of 11 selected genes were determined in all samples in triplicates, and relative expression ratio (R) was calculated with the following formula [47]:

$$ \mathrm{R}=\frac{{{\mathrm{E}}_{\mathrm{target}}}^{{\Delta \mathrm{CP}}_{\mathrm{target}}\left(\mathrm{control}-\mathrm{sample}\right)}}{{{\mathrm{E}}_{\mathrm{reference}}}^{{\Delta \mathrm{CP}}_{\mathrm{reference}}\left(\mathrm{control}-\mathrm{sample}\right)}} $$

## Results

### Anthocyanin profiling

A total of 13 samples were measured for anthocyanin content (Table 2). While only outer or middle taproot tissues were sampled for most of the individuals examined, all three tissue types (outer, middle, inner) were measured for anthocyanin content for two individuals of the CH5544 cultivar sampled at 10 weeks old (Sample A and D). For both samples, the outer purple tissue contained a higher level of anthocyanin compared to the inner purple tissue (Additional file 3). The level of anthocyanin content was the lowest for the middle white tissue for both samples (Additional file 3).

**Table 2:** The type of tissue types sampled for anthocyanin profiling, and the total anthocyanin content for each sample

Sample Name	Individual Name	Cultivar	Tissue type	Tissue sampled	Color	Age	Total anthocyanin content (mg/kg FW)
S1_10AO	A	CH5544	Taproot	Outer	Purple	10 weeks	1220
S2_10AM	A	CH5544	Taproot	Middle	White	10 weeks	35
S3_10AC	A	CH5544	Taproot	Inner	Purple	10 weeks	523
S22_10DO	D	CH5544	Taproot	Outer	Purple	10 weeks	3922
S23_10DM	D	CH5544	Taproot	Middle	White	10 weeks	112
S24_10DC	D	CH5544	Taproot	Inner	Purple	10 weeks	266
S4_10BO	B	CH5544	Taproot	Outer	Purple	10 weeks	359
S19_10CO	C	CH5544	Taproot	Outer	Purple	10 weeks	1202
S20_10CM	C	CH5544	Taproot	Middle	Purple	10 weeks	72
S25_12AO	E	CH5544	Taproot	Outer	Purple	12 weeks	2077
S28_12BO	F	CH5544	Taproot	Outer	Purple	12 weeks	4690
S7_PC_A	G	CH5544	Callus	–	Purple	–	574
S8_PC_B	H	CH5544	Callus	–	Purple	–	381

### RNA sequencing and alignment

After the adaptor and low-quality sequences of pair-end reads were trimmed, a total of 1533 million clean reads were obtained, with an average of ∼34.9 million reads per sample. An average of ∼31 million clean reads per sample, corresponding to ∼89% of the total clean reads were uniquely aligned to the recently published carrot genome [31]. Details on each sample are summarized in Additional file 4.

### Differential gene expression analysis

**Fig. 2:**
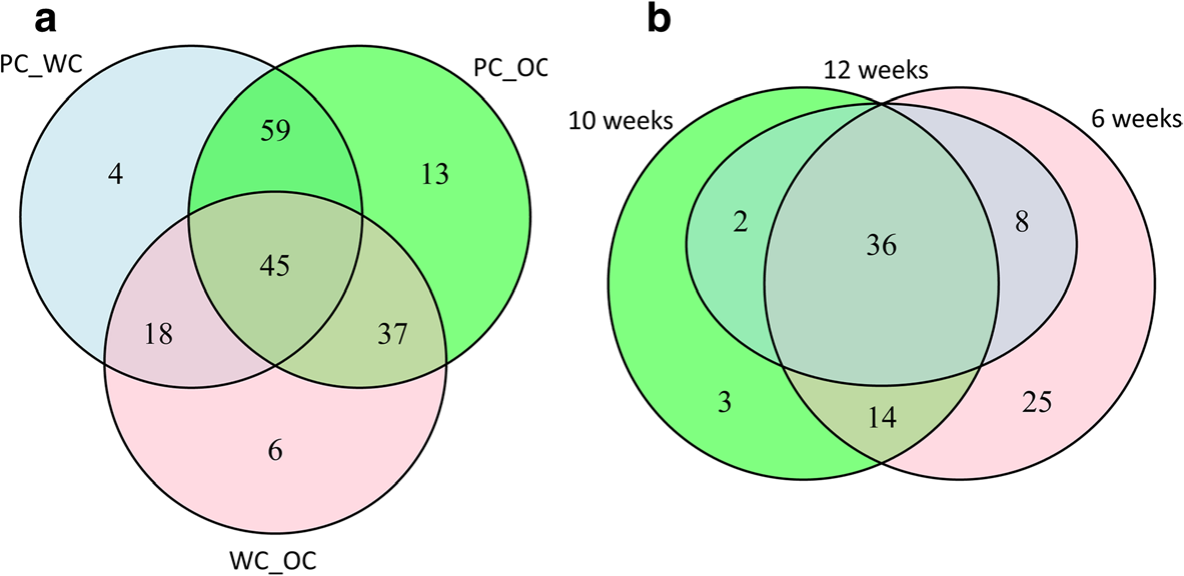
Venn diagram of the differentially expressed genes in carrot calli and taproots. **a** Venn diagram showing the overlap between the differentially expressed genes (DEGs) in the purple, white and orange calli. PC_WC, PC_OC and WC_OC indicate the comparison between purple and white, purple and orange, and white and orange calli, respectively. **b** Venn diagram of the DEGs detected in the comparison between the purple outer and white middle tissue of the CH5544 taproot at the age of 6, 10 and 12 weeks old after sowing

A total of 104 *MYB*-, *bHLH*- and *WD40*-transcription factor genes were identified as differentially expressed at an adjusted *P*-value or FDR < 0.05 when comparing purple, white and orange callus. Specifically, 45 genes were identified as differentially expressed (DE) among all three comparisons, and 59 genes when comparing purple to white and orange calli (Fig. 2a; Additional file 5.1). Of these, 36 genes were identified as DE when comparing the purple outer to middle white tissue in CH5544 sampled at the age of 6-, 10- and 12 weeks old (Fig. 2b; Additional file 5.2). Finally, 11 out of these 36 genes were identified as DE when comparing the purple outer and green/purple inner to middle white tissue in CH5544 sampled at the age of 6-, 10- and 12 weeks old (Additional file 5.2). Some of these genes were identified as DE when comparing the purple outer to white middle/inner tissue in Nightbird, as well as when comparing the purple outer to purple inner tissue in Superblack. Log-fold changes obtained from all 3 Bioconductor packages for all comparisons across various calli and taproot samples are summarized in Additional file 6.

Although the focus of this paper was on MYB, bHLH, WD40 genes, several studies have suggested that bZIP and NAC genes may also play a role in regulating anthocyanin biosynthesis in other plant species [48, 49]. In this study, we have found a small number of genes that seem to be consistently up- or down-regulated in the purple callus and tissue-specific manner across multiple time points. Such results are summarized in Additional file 7.

### Correlation between anthocyanin content and transcriptome abundance for differentially expressed genes

Of a total of 104 *MYB*-, *bHLH*- and *WD40*-transcription factor genes identified as differentially expressed in a purple color-specific manner, 32 genes were significantly correlated with anthocyanin content measured in a subset of callus and taproot samples (Additional file 5.3). A higher proportion of genes were significantly associated with anthocyanin content when genes were identified as DE across multiple comparisons. Specifically, when considering genes identified as DE across comparisons based on calli, as well as outer purple and inner purple/green to middle white tissue for the CH5544 taproot sampled at 6-, 10- and 12 weeks old, a majority of the genes were significantly correlated with anthocyanin content (9 out of 11 genes; Additional file 5.3). When considering genes identified as DE across comparisons based on calli and outer purple to middle white tissue, a smaller proportion of the genes were significantly correlated with anthocyanin content (22 out of 36 genes; Additional file 5.3). Finally, an even smaller fraction of genes were correlated with anthocyanin content when considering genes identified as DE in comparisons using only calli (32 out of 104 genes; Additional file 5.3).

### Comparison of gene expression patterns among various cultivars for genes significantly correlated with anthocyanin content

**Fig. 3:**
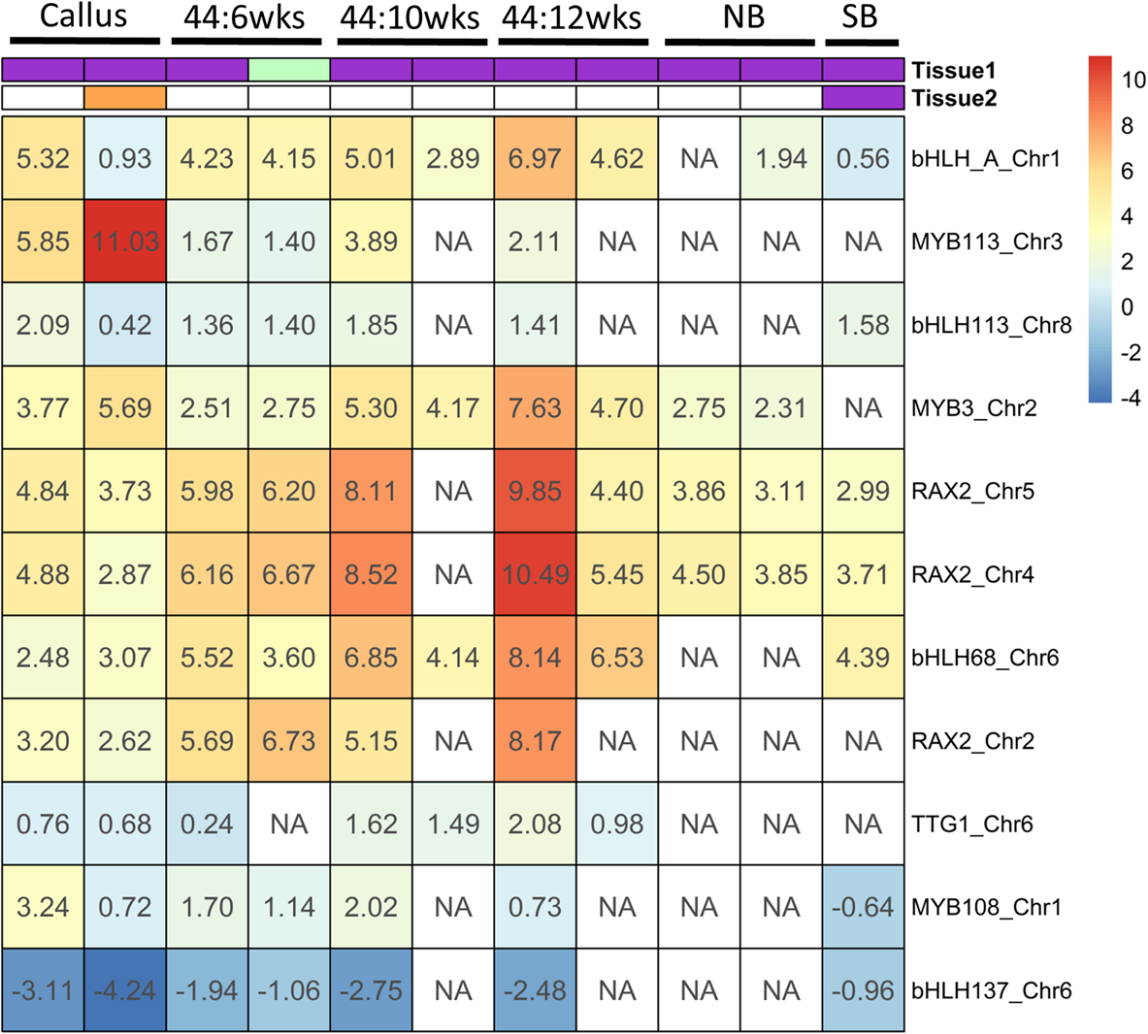
A heatmap of average logFC detected by DESeq2, EdgeR and Limma for 11 transcription factor genes (rows) for which its gene count is significantly correlated with anthocyanin content. Tissue1 and Tissue2 indicate the color of the callus/tissue sample used to perform differential expression analyses. The abbreviation, 44, NB and SB, indicate the cultivar CH5544, Nightbird and Superblack, respectively. For CH5544, the age of the samples are indicated as 6wks, 10wks, and 12wks, indicating 6, 10 and 12 weeks after sowing, respectively. For taproots of each cultivar, the results are summarized as outer/middle and inner/middle tissue comparisons, except for Superblack (SB), in which only outer and inner tissues were compared. A positive or negative number in each cell indicates that a gene was up- or downregulated in purple/slightly green tissue or calli. NA indicates that genes were not differentially expressed by any of the Bioconductor packages used in this study

Of the 32 genes significantly correlated with anthocyanin content, 11 were consistently up- or downregulated in purple/green tissue across various calli and cultivar comparisons (Fig. 3). We were particularly interested in genes that were consistently up- or downregulated in the purple color-specific manner: namely, up- or downregulated in the purple tissue of the CH5544 taproot at 6-, 10- and 12-week-old and the Nightbird taproot, but not strongly differentially expressed in purple outer and purple inner tissue of the Superblack cultivar.

**Fig. 4:**
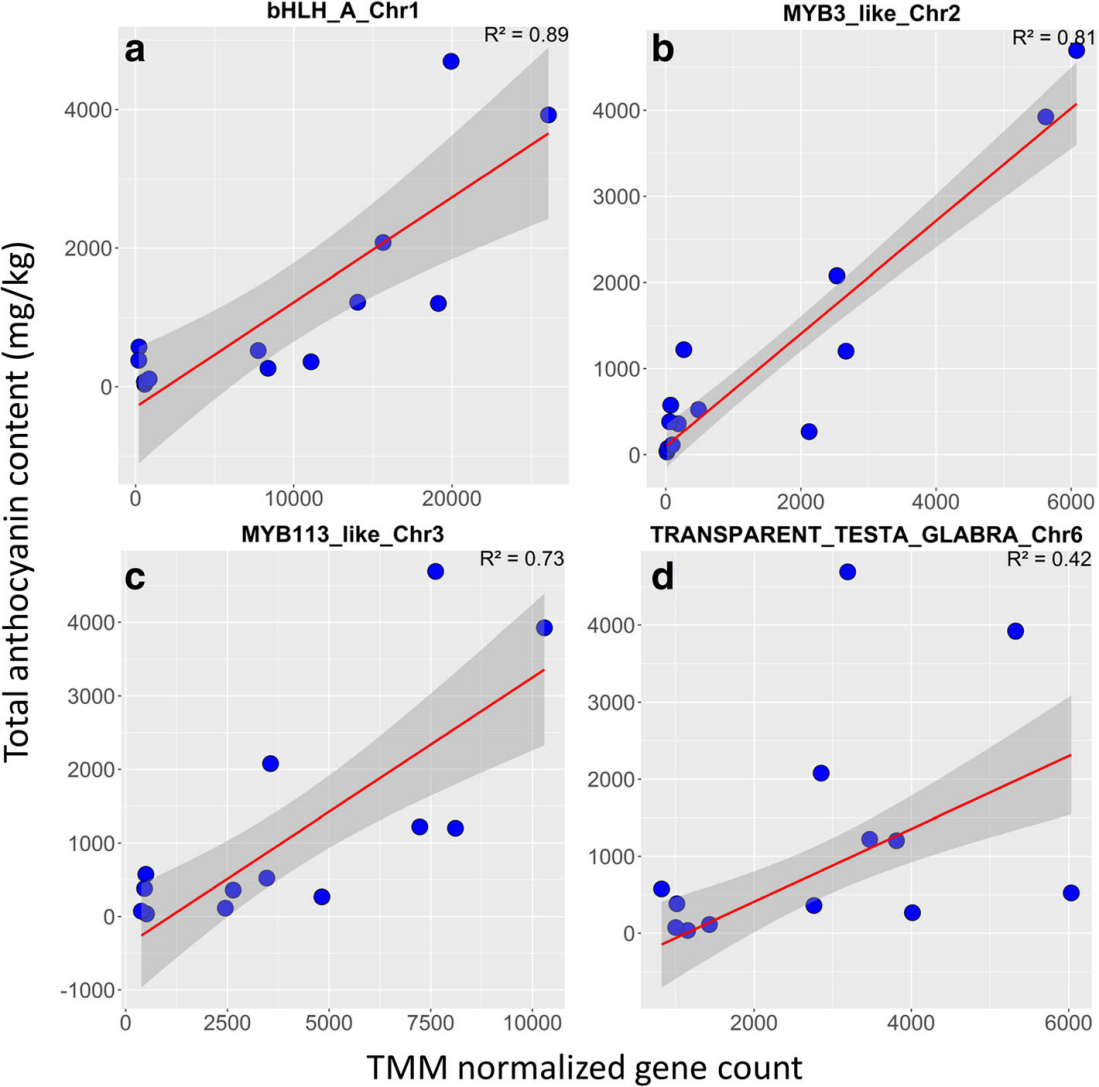
Correlation between the transcriptome abundance and total anthocyanin content for genes differentially expressed in a purple color-specific manner. The red-lines and grey-area represent estimated fit-line and 95% confidence region, respectively. The correlations are showed for: **a** bHLH-A (LOC108204485), **b** MYB3-like (LOC108208100), **c** MYB113-like (LOC108213488), **d** TRANSPARENT TESTA GLABRA 1 (LOC108224236).

*bHLH-A* (LOC108204485) was highly upregulated in purple tissue across almost all comparisons based on calli and taproots of various cultivars (Fig. 3). While the gene was differentially expressed in purple outer and purple inner tissue of the Superblack taproot, its logFC was much smaller compared to other comparisons. The expression of this gene was also significantly positively correlated with anthocyanin content (Fig. 4a).

The *MYB3-like* gene on chromosome 2 (LOC108208100) was consistently upregulated in all purple calli or tissue samples, except for Superblack (Fig. 3; Fig. 4b). The transcriptome abundance of this gene was positively correlated with anthocyanin content. The *RAX2* (*REGULATOR OF AXILLARY MERISTEMS 2*) gene on chromosome 4 (LOC108216892) and *RAX2-like* gene on chromosome 5 (LOC108221019) also exhibited similar patterns, although these genes were differentially expressed in Superblack.

The *MYB113-like* gene, also known as *DcMYB6*, was previously demonstrated to be involved in regulating anthocyanin biosynthesis in purple carrot taproots [17]. In the present study, the gene was also differentially expressed in a purple color-specific manner, although it was not detected as DE in Nightbird. The transcriptome abundance of this gene was significantly positively correlated with anthocyanin content (Fig. 4c). Similarly, *TRANSPARENT TESTA GLABRA 1* (*TTG1*; LOC108224236) was also upregulated in the purple callus and purple tissue of the CH5544 cultivar across all ages, however it was not differentially expressed in Nightbird. The transcriptome abundance of this gene was also positively correlated with anthocyanin content (Fig. 4d).

### Validation of the detected candidate genes with qRT-PCR

**Fig. 5:**
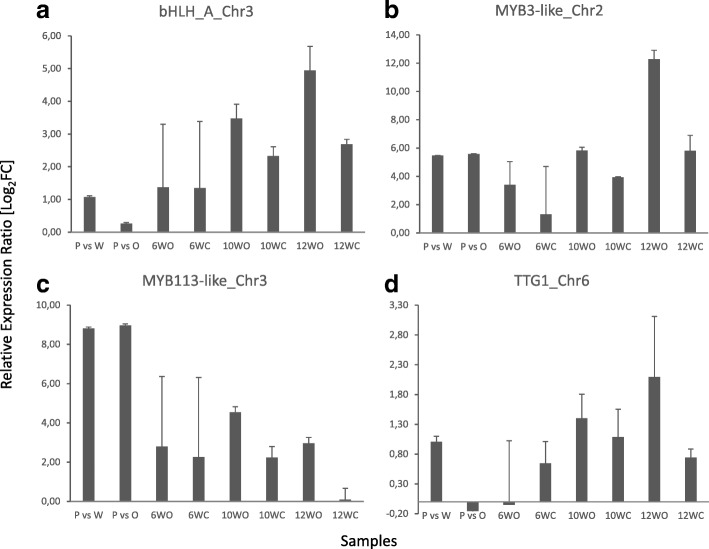
Validation of differentially regulated genes associated with the anthocyanin content by qRT-PCR. Data were normalized to the expression of glyceraldehylde 3-phosphate dehydrogenase (G3PDH) for each sample. P vs W indicates the comparison between purple and white calli, and P vs O indicates the comparison between purple and orange calli. 6WO, 10WO and 12WO indicate the comparison between outer and middle tissue for 6-, 10- and 12-week-old taproots, respectively. Similarly, 6WC, 10WC and 12WC indicate the comparison between center and middle section for 6-, 10- and 12-week-old taproots, respectively. The results are showed for the following genes: **a** bHLH-A (LOC108204485), **b** MYB3-like (LOC108208100), **c** MYB113-like (LOC108213488), **d** TRANSPARENT TESTA GLABRA 1 (LOC108224236).

Relative Expression Changes based on qRT-PCR results were largely found to be in accordance to the RNA-Seq data, although with a few exceptions. In particular, *bHLH-A* (LOC108204485; Fig. 5a), *MYB3-like* (LOC108208100; Fig. 5b), *MYB113-like* (LOC108213488; Fig. 5c), *RAX2-like* (LOC108221019), *RAX2* (LOC108216892), *RAX2-like* (LOC108208253) and *bHLH113-like* (LOC108197411) were highly upregulated across all purple callus/tissue samples, and the relative expression ratio increased in a color and age-specific manner. In a similar manner, *bHLH137-like* (LOC108225152) was consistently downregulated in purple callus and tissues, validating the results of the differential expression analyses. The *TTG 1* gene (LOC108224236; Fig. 5d) was also found to be slightly upregulated in a color and age specific manner across all comparisons, except for the comparison between purple and orange callus. *bHLH68* (LOC108213035) expression was found to be upregulated in purple as compared to orange callus, and outer purple tissue of all 6 and 12 weeks old taproots as compared to middle tissue; however, this gene was slightly downregulated in outer of 10 and center of 12 weeks old taproots as compared to the middle white tissue. Finally, *MYB108* (LOC108200913) was consistently downregulated in purple callus, but upregulated in outer and center purple tissue of CH5544 in later stages. These results are summarized in Additional file 2.3.

## Discussion

Our study aimed to identify important genes that function as positive or negative regulators in the anthocyanin biosynthesis pathways in carrots using RNA-Seq and qRT-PCR for validation. RNA was extracted from differently colored calli, as well as tissue samples from taproots of various cultivars across the course of development. Using the recently published carrot genome [31], gene expression levels of these samples were compared, and *MYB*, *bHLH* and *WD40* genes that were consistently up- or downregulated in a purple color-specific manner were identified. In total, 104 *MYB*, *bHLH* and *WD40* genes were differentially expressed. Using anthocyanin content measured from a subset of calli and tissue samples, the expression of 32 genes (out of 104 genes) were shown to be significantly correlated with anthocyanin content. Expression patterns of these genes were compared across various cultivars and different time points, and 11 genes (out of 32 genes) were consistently up- or downregulated in a purple color-specific manner. Finally, we validated 10 out of these 11 genes using qRT-PCR, demonstrating that these regulatory genes may indeed activate or hinder anthocyanin biosynthesis in black carrot.

Our study has demonstrated that the *R2R3-MYB* transcription factor, *MYB113-like* gene on chromosome 3 (LOC108213488), was upregulated in a purple color-specific manner, and the expression of this gene was strongly positively correlated with anthocyanin content. This supports the previous findings of Xu et al. [17], that found that this gene (designated as *DcMYB6*) is involved in regulating anthocyanin biosynthesis in purple carrots. This gene contains the highly conserved *bHLH*-interaction motif and two atypical motifs of anthocyanin regulators, and shares high identity with anthocyanin-regulating *MYB* transcription factors from many other species [17].

Our study also demonstrates that the basic helix-loop-helix protein A (*bHLH-A*; LOC108204485), which is homologous to *TT8* in *Arabidopsis*, may act as a positive regulator in anthocyanin biosynthesis. To our knowledge, there is no documentation for the role of this gene in carrot anthocyanin biosynthesis. The *bHLH-A* gene has been shown to be upregulated in eggplant (*Solanum melongena L.*) peel, indicating that this gene may enhance anthocyanin biosynthesis [50]. Hellens et al. [51] identified that pea gene A, which is the factor determining anthocyanin pigmentation in pea (*Pisum sativum*), was closely related to the *MtbHLHA* gene in *Medicago truncatula*; this study also found that gene A and *MtbHLHA* are in the same clade as *AN1* in petunia and *TT8* in *Arabidopsis*, both of which are known to directly activate transcription of structural genes in the anthocyanin biosynthesis pathway [15, 52]. More recent studies found genes that are closely related to *AN1* and *TT8* also act as a positive regulator for anthocyanin biosynthesis in red radish (*Raphanus sativus L.*) [53] and ornamental cabbage (*Brassica oleracea var. acephala*) [54]. In a separate study, we overexpressed *AmDEL* (bHLH), together with *AmRosea1* (R2R3-MYB) from *Antirrhinum majus* in orange carrot cultivar Danvers 126 [55]. These transcription factors (TFs) are known to affect anthocyanin pigmentation of snapdragon flowers, and ectopic expression of these TFs has been reported to induce anthocyanin biosynthesis in *Solanum lycopersicum* [56, 57]. The simultaneous expression of both of these TFs led to synthesis and accumulation of cyanidin-based anthocyanins in calli, shoots and taproots of orange carrot, whereas individual overexpression of either of these TFs did not result in any pigmentation. Our study confirms the presence of intact biosynthetic genes responsible of anthocyanin biosynthesis in orange carrots and absence of anthocyanins could only be attributed to a lack of necessary transcription factors. Furthermore, the inability of *AmRosea1* and *AmDEL* in inducing anthocyanin biosynthesis individually confirms the requirement of formation of *MYB*-*bHLH*-*WD40* (MBW) complex for activation of anthocyanin biosynthesis genes.

*TRANSPARENT TESTA GLABRA 1* on chromosome 6 (*TTG1*; LOC108224236) was also expressed in a purple color-specific manner, and its expression was positively correlated with anthocyanin content in the current study. A homolog to this gene is known to encode a *WD40* repeat protein in *Arabidopsis* [19]*.* Together with *bHLH* and *MYB* proteins, *WD40* repeat proteins are required to form the *MYB*-*bHLH*-*WD40* (MBW) transcriptional complex and activate the anthocyanin biosynthetic pathway. However, unlike *bHLH* or *MYB* proteins, *WD40* proteins seem to have a more general role in the regulatory complex [58], and the *TTG1* gene was expressed in its all major organs of *Arabidopsis* [19]. In the current study, while the *TTG1* gene was consistently differentially expressed in a purple color-specific manner, its log-fold change was generally low (Fig. 3). This, in combination with its moderate correlation to anthocyanin content, suggests *WD40* may not be as crucial as the *bHLH* or *MYB* proteins detected in this study, although *WD40* proteins are still required to activate the structural genes involved in the anthocyanin biosynthesis.

The results of the current study indicate that a total of three *RAX2* (*REGULATOR OF AXILLARY MERISTEMS 2*) and *RAX2-like* genes may also function as positive regulators for anthocyanin biosynthesis. *RAX* genes are involved in shoot branching in *Arabidopsis* [59, 60]. In particular, *RAX2* controls axillary meristem formation during middle to late stages of vegetative development [60]. All *RAX* genes are known to belong to the class *R2R3 MYB* family [59, 61]. However, the role of these genes on anthocyanin biosynthesis in carrots or any other plant species is currently unknown, and future efforts should be directed towards understanding the function of these genes in anthocyanin production.

The *bHLH137-like* gene on chromosome 6 (LOC108225152) was consistently downregulated, and its expression was negatively correlated with anthocyanin content, indicating that this gene may act as a downregulator in the anthocyanin pathways. Currently there is no documentation of its role in anthocyanin biosynthesis in the carrot or any other root species. In *Arabidopsis*, the *bHLH137* gene was reported as a DELLA-induced gene that may repress signaling of phytohormone gibberellic acid, and this gene is predicted to encode transcription factors [62]. In other species, the role of this gene is not well understood. *bHLH137* in *Arabidopsis* was shown to be closely related to genes involved in growth regulation, including the grapevine (*Vitis vinifera*) cell elongation *bHLH* protein (*VvCEB1*) [63]; there is a conflicting evidence that *VvCEB1* may or may not play a role in cell expansion during berry development for grapevine [63, 64].

Finally, the *MYB3-like* gene on chromosome 2 (LOC108208100) was also shown to be upregulated in a purple color-specific manner across all time points and cultivars except for Superblack, and it was strongly correlated with anthocyanin content. *MYB3* is a *MYB* transcription factor that is possibly involved in phenylpropanoid metabolism in *Arabidopsis* [65]; phenylpropanoids are a diverse group of compounds involved in plant defense, structural support, and survival, and the anthocyanin biosynthetic pathway is a major branch of the phenylpropanoid pathway. To our knowledge, the role of *MYB3* is not well understood in carrot or any other root species.

## Conclusions

We have uncovered possible candidate genes that may regulate the anthocyanin biosynthesis pathways in black carrot. Transcriptomic data were obtained from differently colored calli, as well as taproot samples of various cultivars across the course of development. A total of 10 *MYB*, *bHLH* and *WD40* genes were consistently up- or downregulated in a purple color-specific manner; the expression of these genes was significantly correlated with anthocyanin content, and the expression results were validated with qRT-PCR. Our results provide insights into regulatory genes that may be responsible for anthocyanin production in carrot, and suggest that future efforts could be directed towards understanding how these candidate genes regulate the anthocyanin biosynthesis pathways in this species.

## Additional files

Additional file 1: Cross section of carrot taproot. (TIF 5480 kb)

Additional file 2: Sequence and primer properties used to analyses 11 candidate genes and GAPDH (Additional file 2.1), PCR efficiencies calculated for primer pairs using 4 fold (based on mean Ct values observed for triplicates) dilution series of cDNA mix from all 30 samples (Pfaffl 2001; Additional file 2.2), relative expression ratio of 11 candidate genes in purple vs non-purple calli/tissue (Average of biological samples) calculated with following formula (Pfaffl 2001; Additional file 2.3); trimmed mean of M-values (TMM) of 11 candidate genes in purple and non-purple calli/tissue (Additional file 2.4). (XLSX 58 kb)

Additional file 3: Anthocyanin content for outer, middle and inner tissues of two CH5544 samples obtained at 10 weeks after sowing. (TIFF 23100 kb)

Additional file 4: Sample name, sample type (callus or tissue), age of the sample, as well as the number of clean reads, the number of uniquely mapped reads and the percentage of uniquely mapped reads for each sample. Texts in parentheses under “Callus” or “Tissue” indicate the color of the sample. (XLSX 13 kb)

Additional file 5: The results from the differentially expression analyses using callus (Additional file 5.1), using callus and taproots of all cultivars (Additional file 5.2), the results from the linear regression analyses testing for the significant association between the gene count and anthocyanin content (Additional file 5.3). (XLSX 663 kb)

Additional file 6: This file contains the results from differential expression analyses using DESeq2, EdgeR (glm model), EdgeR (QL model) and Limma. Each sheet contains results from callus (1.Callus), as well as taproots of 6 weeks old CH5544 (2.CH5544_6wks), 10 weeks old CH5544 (3.CH5544_10wks), 12 weeks old CH5544 (4.CH5544_12wks), Nightbird (5.Nightbird) and Superblack (6.Superblack). For all sheets, results are organized as: GeneID, chromosome, start position of the gene, end position of the gene and the gene product. (XLSX 28300 kb)

Additional file 7: The results from differential expression analyses for NAC and bZIP genes using DESeq2, EdgeR (glm model), EdgeR (QL model) and Limma. Results are organized as GeneID, chromosome, start position of the gene, end position of the gene, the gene product, and the number of programs that identified the gene as differentially expressed for the pairwise comparisons in the following order: callus, taproots of 6 weeks old CH5544, 10 weeks old CH5544, 12 weeks old CH5544, Nightbird and Superblack. (XLSX 21 kb)
