# Supplementary figures and images for: Identification of transcription factor genes involved in anthocyanin biosynthesis in carrot (Daucus carota L.) using RNA-Seq
Source: BMC Genomics. 2018 Nov 8;19:811. doi: 10.1186/s12864-018-5135-6 (PMC6225646; doi:10.1186/s12864-018-5135-6)

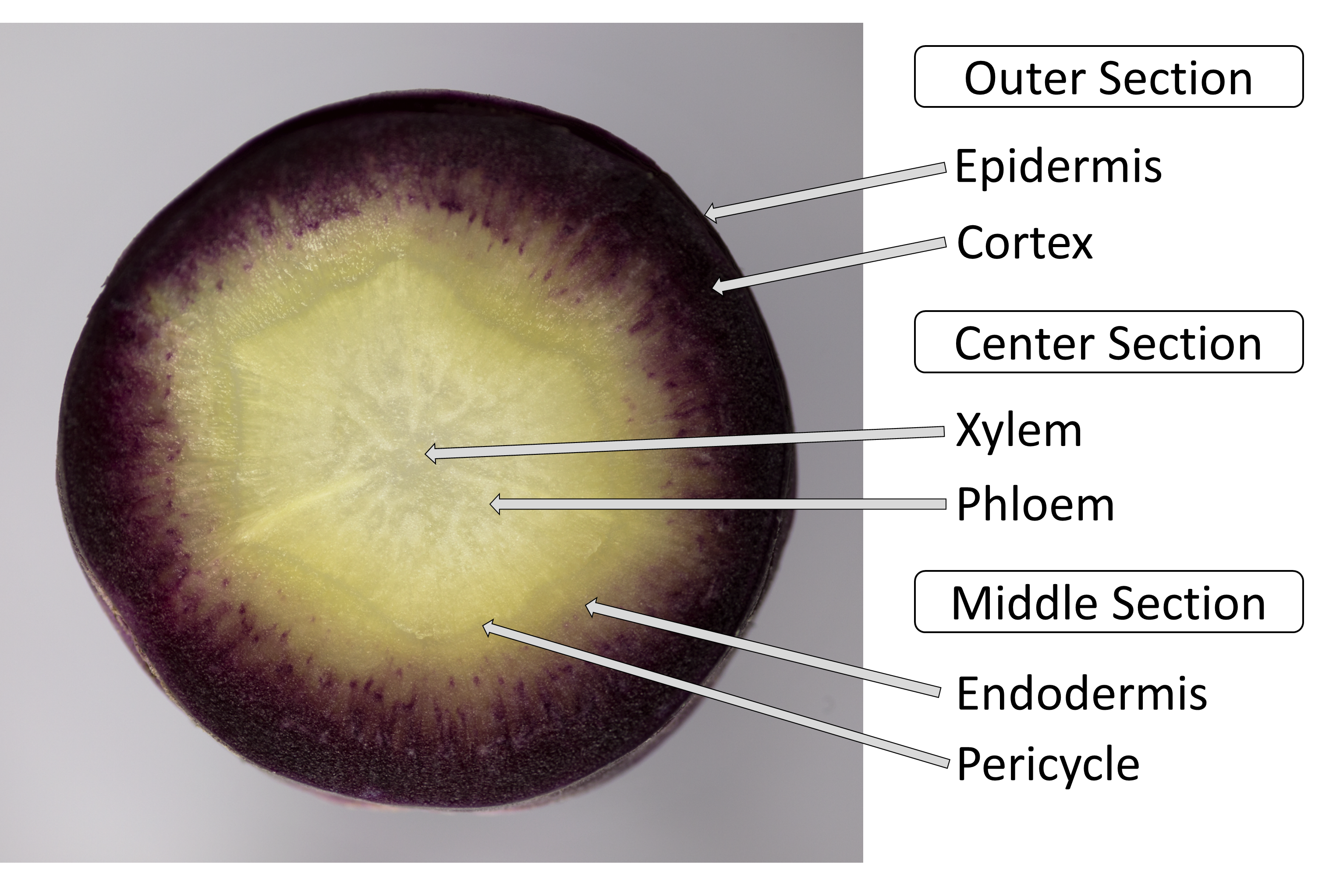

Supplement: Supplementary file 1 — Cross section of carrot taproot. (TIF 5480 kb) [file 12864_2018_5135_MOESM1_ESM.tif]
